# Divergent Paths for Adult Mortality in Russia and Central Asia: Evidence from Kyrgyzstan

**DOI:** 10.1371/journal.pone.0075314

**Published:** 2013-10-08

**Authors:** Michel Guillot, Natalia Gavrilova, Liudmila Torgasheva, Mikhail Denisenko

**Affiliations:** 1 University of Pennsylvania, Philadelphia, Pennsylvania, United States of America; 2 University of Chicago, Chicago, Illinois, United States of America; 3 National Statistical Committee of the Kyrgyz Republic, Bishkek, Kyrgyzstan; 4 National Research University “Higher School of Economics”, Moscow, Russia; London School of Economics, United Kingdom

## Abstract

Adult mortality has been lower in Kyrgyzstan vs. Russia among males since at least 1981 and among females since 1999. Also, Kyrgyzstan’s mortality fluctuations have had smaller amplitude. This has occurred in spite of worse macro-economic outcomes in Kyrgyzstan. To understand these surprising patterns, we analyzed cause-specific mortality in Kyrgyzstan vs. Russia for the period 1981-2010, using unpublished official data. We find that, as in Russia, fluctuations in Kyrgyzstan have been primarily due to changes in external causes and circulatory causes, and alcohol appears to play an important role. However, in contrast with Russia, mortality from these causes in Kyrgyzstan has been lower and has increased by a smaller amount. As a result, the mortality gap between the two countries is overwhelmingly attributable to external and cardio-vascular causes, and more generally, to causes that have been shown to be strongly related to alcohol consumption. These cause-specific results, together with the existence of large ethnic differentials in mortality in Kyrgyzstan, highlight the importance of cultural and religious differences, and their impact on patterns of alcohol consumption, in explaining the mortality gap between the two countries. These findings show that explanatory frameworks relying solely on macro-economic factors are not sufficient for understanding differences in mortality levels and trends among former Soviet republics.

## Introduction

A puzzling pattern of mortality trends in the former Soviet republics of Central Asia is that declines in life expectancy in the years following the break-up of the Soviet Union have not been as large as in Russia. For example, in Kyrgyzstan – the Central Asian republic that is the focus of this paper -, reported life expectancy at birth for both sexes combined declined by 3.3 years between 1990 and 1995 (from 68.8 to 65.5 years), while in Russia during the same period, it declined by 4.6 years (from 69.3 to 64.7 years). Moreover, for males, official estimates show that life expectancy has been consistently higher in Kyrgyzstan since the early 1990s. The most recent estimates show that, in 2009, male life expectancy remains higher in Kyrgyzstan by about 2 years [1].

This is puzzling to many observers, because Kyrgyzstan is a much poorer country than Russia, and in many ways has been more severely hit by the economic crisis that followed the collapse of the Soviet Union in 1991. Starting from a much disadvantaged standpoint in 1990, Kyrgyzstan underwent faster declines in gross domestic product (GDP) per capita during the first half of the 1990s and did not experience the same kind of rapid economic growth that Russia experienced in the 2000s. Between 1990 and 2010, the Russia/Kyrgyzstan ratio in GDP per capita increased from 4.4 to 8.9 [2]. Unemployment information also shows larger deteriorations in Kyrgyzstan in the 1990s [3]. Since mortality fluctuations in Russia and other countries of the former Soviet Union have been primarily attributed to abrupt changes in the economic situation, including drops in GDP per capita and increases in unemployment [3-9], similar – if not greater – mortality increases should be expected in a Central Asian republic like Kyrgyzstan.

The most immediate explanation is that data quality may be lower in Central Asia and may have deteriorated since the break-up of the Soviet Union, while in Russia official mortality rates may have reflected true patterns [10]. This would have created a situation in which official mortality rates in Central Asia are too low and underestimate the scale of the mortality deterioration. Indeed, data quality has always been described as problematic in Central Asia [11]. However, recent evaluations of data quality in Kyrgyzstan have brought qualifications to this statement. The conclusion is that the amount of underestimation varies greatly over time and age. While reported infant mortality is significantly underestimated and shows clear signs of deterioration in the 1990s [12], reported adult mortality appears to provide a reliable picture of true mortality levels and trends at these ages, especially since the 1980s [13-15].

**Figure 1:**
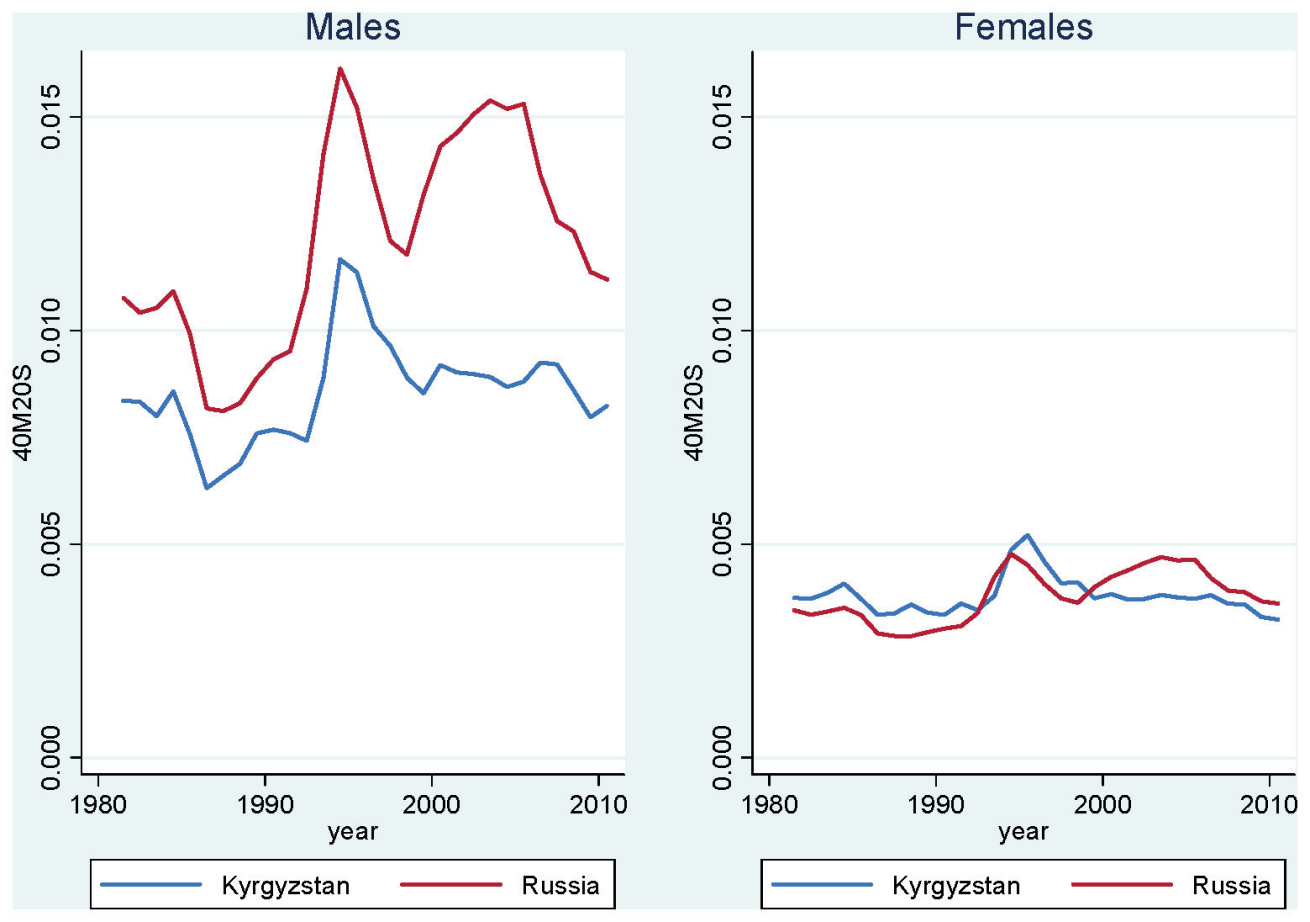
Age-Standardized Mortality Rate at Ages 20-59, Kyrgyzstan and Russia, 1981-2010. Source: Kyrgyzstan: Official population estimates and death registration tables (Forms 5 [1981-87] and S51 [1988-2010]). Russia: Human Mortality Database [19] for population estimates; Meslé et al. [18] for deaths in 1981-88; Official death registration tables (Forms S51) for deaths in 1989-2010.

Interestingly, the mortality patterns described earlier for life expectancy hold when we focus on these more reliable adult ages. Figure 1 shows age-standardized death rates at ages 20-59 in Kyrgyzstan vs. Russia, by sex, for the period 1981-2010. Among males, we see that adult mortality has been systematically lower in Kyrgyzstan. We also see that mortality fluctuations have occurred in both countries in a similar fashion: abrupt mortality decline in 1985-1987, related to Gorbachev’s anti-alcohol campaign, and abrupt mortality increase after 1991, following the collapse of the Soviet Union. However, the amount of fluctuation has been smaller in Kyrgyzstan, both in absolute and relative terms. We also see that starting in 1998, while adult mortality increased sharply again in Russia (an increase that has been associated with the collapse of the ruble [16]), it remains more or less constant in Kyrgyzstan. Overall, we observe an increase in the gap between the two countries during the 1990s and the first half of the 2000s. Although the gap has narrowed recently, adult mortality is still 1.30 times greater in Russia. This ratio is larger than it was in 1990, on the eve of the break up of the Soviet Union. For females, adult mortality was actually higher in Kyrgyzstan during the 1980s and 1990s. However, Russian females lost their advantage in 1999. As of 2010, adult mortality is higher in Russia for both males and females.

In this paper, we investigate the reasons for Kyrgyzstan’s lower adult mortality and smaller fluctuations by examining the causes of death that have generated these patterns. We first examine the causes of death explaining trends in adult mortality in Kyrgyzstan. We then estimate the causes of death that are responsible for the difference in adult mortality between Kyrgyzstan and Russia. Finally, in light of these results, we evaluate existing explanations that have been proposed for interpreting divergent mortality patterns among former Soviet republics.

## Materials and Methods

This paper relies for the most part on official mortality data from both Kyrgyzstan and Russia. For Kyrgyzstan, information on deaths by cause comes from unpublished aggregate tabulations of deaths by age, sex and detailed cause (Forms No. 5 and S51). These tables contain the original and most detailed cause-of-death information available for Kyrgyzstan. Information available in the WHO Mortality Database [17], although derived from the same original information, does not contain the level of detail in terms of causes of death necessary for some of the analyses performed in this paper. Population estimates for Kyrgyzstan are official annual population estimates by age and sex. Both population and deaths data for Kyrgyzstan were obtained directly from the National Statistical Committee (NSC) of the Kyrgyz Republic. To our knowledge, this is the first time that cause-of-death information in Kyrgyzstan is analyzed with such level of detail. For Russia, we use official deaths data for the period 1989-2010. For the period 1981-1988, we rely on death data provided by Meslé et al. [18]. Russia’s population estimates by age and sex are taken from the Human Mortality Database [19].

We focus here on two different groupings of causes of death. In a first set of analyses, we use the seven broad causes commonly used in analyses of cause-specific mortality: (1) Infectious diseases; (2) Neoplasms; (3) Circulatory diseases; (4) Respiratory diseases; (5) Digestive diseases; (6) External causes; and (7) All other causes (including ill-defined causes).

In a second set of analyses, we examine causes of death that have been identified as strongly-related to alcohol consumption in several studies based on Russian data. The reason for this particular focus on alcohol-related mortality is that these causes have been shown to explain most of the fluctuations in Russian mortality [8,16,20]. Since all-cause adult mortality in Kyrgyzstan presents both similar and contrasting features with Russia, we would like to examine the role of alcohol-related mortality in explaining these similarities and differences.

The identification of strongly alcohol-related causes is based on a case-control study of adult deaths in three cities of Western Siberia [16]. Causes were identified as strongly alcohol related if they presented a strong dose-response association with alcohol consumption. (The decedent’s past alcohol use was provided by family proxy information.) These causes include: upper aero-digestive tract cancer; tuberculosis; pneumonia; liver disease other than cancer; pancreatic disease other than cancer; heart disease other than myocardial infarction; ill-specified causes; alcoholic-related disorders, including alcohol psychosis and chronic alcoholism; and external causes, including alcoholic poisoning. Obviously not all the deaths from these causes are due to alcohol. The purpose of this grouping is not to estimate the amount of mortality attributable to alcohol, but to examine the extent to which adult mortality trends in Kyrgyzstan vs. Russia can be explained by causes that have been shown to be strongly related to alcohol in Zaridze’s study [16] vs. causes that have been shown not to be strongly related to alcohol.

The classification of deaths by cause changed over time in both countries, but in different ways. Before the break-up of the Soviet Union, codes where similar in both countries. Discrepancies started to appear after independence. Most notably, Russia adopted a modified version of ICD-10 in 1999, with its own set of numerical codes (1-238). In Kyrgyzstan, the transition to ICD-10 occurred in 2000, but with the use of ICD-10 alpha-numerical codes (A00-Y98). Given the broad grouping of causes that we use in this paper, changes of classification were relatively straightforward to accommodate in our analyses. The codes we use are presented in Table S1 (for Kyrgyzstan) and S2 (for Russia).

We focus on adult ages (20-59) because this age range has emerged as critical for understanding mortality fluctuations in Soviet and post-Soviet states [21]. For males, this age range also explains a very large portion of the gap in life expectancy at birth between Kyrgyzstan and Russia. A decomposition of life expectancy at birth (results not shown) tells that mortality in this age range explains a minimum of 84% of the gap during our period of interest. In fact, for some of the years this age range explains more than 100% of the life expectancy gap between the two countries, due to the fact that Kyrgyzstan’s actual life expectancy advantage is somewhat attenuated by its higher levels of child mortality [12]. For females, starting in 1999, adult mortality contributes positively to the difference in life expectancy between Kyrgyzstan and Russia, even though for most years life expectancy in Russia has remained slightly higher, here again due to Kyrgyzstan’s higher levels of infant and child mortality. Another reason for focusing on ages 20-59 is that this age range appears to be less affected by data quality problems than other age groups such as infant and child ages. Our focus on adult ages is thus motivated by both substantive and practical reasons. We standardize mortality in this age range using equal weights for each five-year age group. Thus our age-standardized mortality rate, _40_M_20_^S^, is equal to 1/8 · ( _5_M_20_ + _5_M_25_ + … + _5_M_55_). The age-standardized mortality rate by cause is calculated in the same manner, using cause-specific instead of all-cause deaths in the numerator of the rates. These cause-specific age-standardized mortality rates add up to the age-standardized rate for all causes combined. Therefore the contribution of cause-specific mortality to differences in all-cause _40_M_20_^S^ over time and place can be directly estimated by examining differences in cause-specific _40_M_20_^S^.

Kyrgyzstan is a multi-ethnic society. While the ethnic Kyrgyz represent the largest ethnic group, there are substantial Russian and Uzbek minorities. The ethnic distribution of the population changed rapidly during the 1990s. In particular, many ethnic Russians left Kyrgyzstan to return to Russia. Between 1989 and 1999, the percentage of ethnic Russians declined from 21.5% to 12.5%. If we focus on the age group 20-59 for the same period, the percentage of ethnic Russians declined from 24.0% to 13.5% for males, and from 25.8% to 15.3% for females. By 2009, these percentages declined to 8.0% for males and 9.4% for females. These compositional changes affect mortality trends at the national level, because ethnic Russians and ethnic Kyrgyz exhibit quite distinct mortality profiles [14,15]. Unfortunately, deaths by cause and ethnicity, together with population by ethnicity, are not available on an annual basis, preventing the calculation of ethnic- and cause-specific mortality trends. In this paper, we focus on national-level mortality trends, keeping in mind that these trends reflect in part changes in the ethnic composition of the population. The importance of ethnicity for understanding mortality patterns in the region is discussed at the end of the paper.

## Results

### Trends in cause-specific adult mortality in Kyrgyzstan, 1981-2010

**Figure 2:**
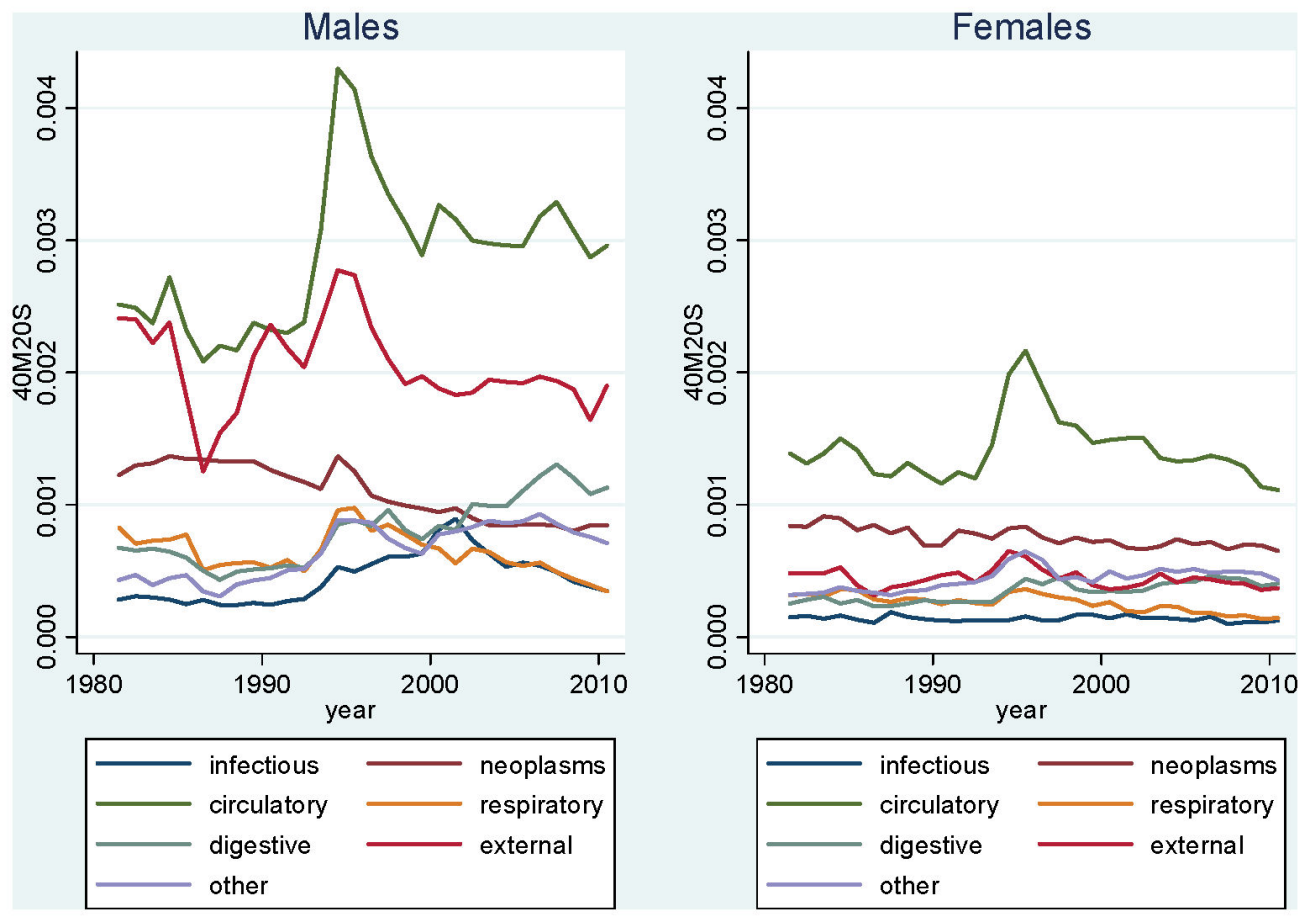
Age-Standardized Mortality Rate at Ages 20-59, Kyrgyzstan, 1981-2010, by Broad Cause. Source: Official population estimates and death registration tables (Forms 5 [1981-87] and S51 [1988-2010]).

Figure 2 shows trends in cause-specific mortality (broad causes) in Kyrgyzstan between 1981 and 2010. For males, we find that fluctuations in all-cause mortality are primarily explained by changes in circulatory diseases and external causes. While both sets of causes play an important role in these fluctuations, we find that external causes play a more important role in the fluctuations associated with Gorbachev’s anti-alcohol campaign (1984-89). The large fluctuations of the 1990s, however, are primarily explained by changes in mortality from circulatory diseases. Indeed, starting from a similar level in the early 1990s, mortality from circulatory diseases increased by a larger amount than mortality from external causes between 1992 and 1995. The other five broad causes presented in Figure 2 play a much smaller role overall, and do not explain much of the mortality fluctuations. Infectious diseases increased substantially during the 1990s, but have decreased during the 2000s. We note an important increase in digestive diseases since the early 1990s, which now rank as the third highest broad cause among males.

For females, fluctuations are primarily explained by variations in circulatory diseases. External causes play some role in explaining the 1984-89 fluctuation, but this role is less important than that of circulatory causes. Overall, the role of external causes is much smaller than for males. Cancers is the second cause of death among adult females in Kyrgyzstan, but played little role in the mortality fluctuations. The other broad causes also remained more or less constant throughout the period.

Many deaths within the external broad category are known to be related to alcohol consumption, most notably deaths from alcohol poisoning. Also, it has been shown that, in Russia, many alcohol-related deaths are wrongly attributed to diseases of the circulatory system, especially to heart diseases other than myocardial infarction [20]. Given that these two broad causes, like in Russia, explain most of the fluctuations in adult mortality in Kyrgyzstan, we suspect that alcohol plays an important role in Kyrgyzstan as well. In order to further examine the role of alcohol, we thus calculated mortality from causes identified as strongly-related to alcohol in Zaridze’s study, as explained in the previous section.

**Figure 3:**
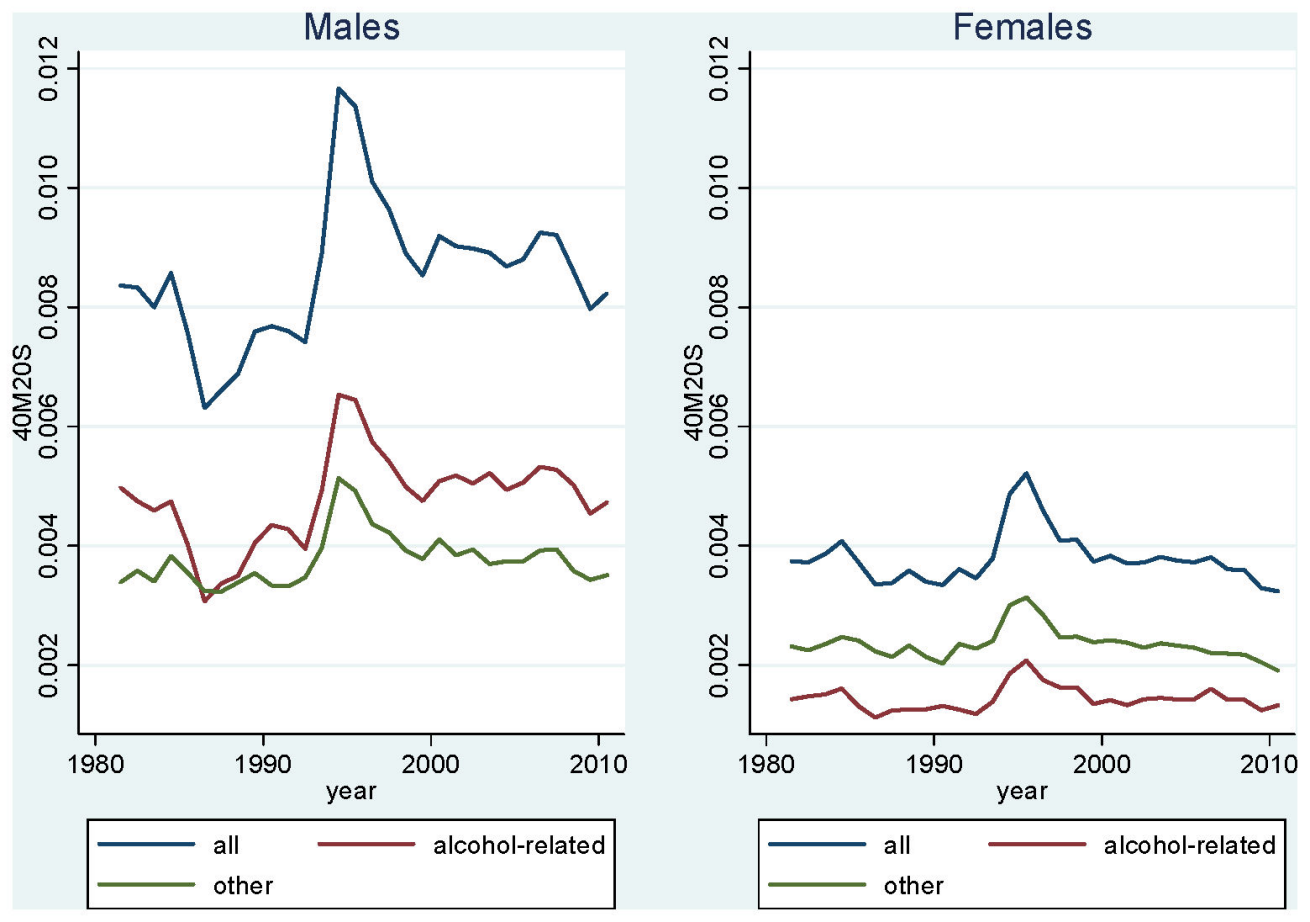
Age-Standardized Mortality Rate at Ages 20-59, Kyrgyzstan, 1981-2010, All Causes and Strongly Alcohol-Related Causes. Source: Official population estimates and death registration tables (Forms 5 [1981-87] and S51 [1988-2010]).

Figure 3 shows trends in strongly alcohol-related adult mortality in Kyrgyzstan, together with mortality from all other causes. For males, we find that alcohol-related causes dominate throughout the period. However, there is a clear distinction between the 1984-89 and 1992-99 fluctuations. The 1984-89 fluctuation is almost entirely explained by mortality attributable to alcohol-related causes. Indeed, mortality from other causes varied little during this period. This is expected given the context of this anti-alcohol campaign, which by nature should affect causes directly or indirectly related to alcohol, while having little or no effect on other causes. In 1992-99, we find that most of the fluctuation is explained by alcohol-related causes. However, we also find a substantial increase in mortality from other causes.

For females, we find that, in contrast to males, “other causes” dominate throughout the period. However, the respective role of alcohol-related vs. other causes in explaining the fluctuations is similar to males. The 1984-89 fluctuation is almost entirely explained by alcohol-related causes, while the 1992-99 fluctuation is explained by both sets of causes, with a somewhat larger increase for alcohol-related causes.

Overall, this analysis of cause-specific mortality in Kyrgyzstan presents many similarities with patterns observed in Russia. Fluctuations occurred at about the same time, and are explained by the same broad causes, namely, external causes and circulatory causes [6,20,22]. Like in Russia, alcohol seems to play an important role in these fluctuations [16,23,24]. These similarities are not very surprising, given the fact that Kyrgyzstan was one of the most russified Central Asian republics, and in many ways has experienced similar socio-economic changes since independence. Kyrgyzstan remains strongly anchored in the former Soviet space, and in many areas Kyrgyzstan’s experience parallels that of Russia.

### Cause-specific contributions to the mortality gap between Russia and Kyrgyzstan

In spite of the similarities highlighted above, adult mortality in Kyrgyzstan vs. Russia also exhibit important discrepancies, as discussed above. First of all, adult mortality is lower in Kyrgyzstan among males throughout the period, and among females since 1999. We also find larger mortality fluctuations in Russia.

The analysis of broad causes in both Russia and Kyrgyzstan (results not shown) tells us that, for males, the gap between the two countries is primarily explained by external causes, with circulatory causes also playing an important role in 1998-2010. For females, the loss of the Russian advantage is primarily explained by mortality from external causes, which increased by a larger amount in Russia, and by mortality from circulatory causes, which became higher in Russia in the early 2000s.

**Figure 4:**
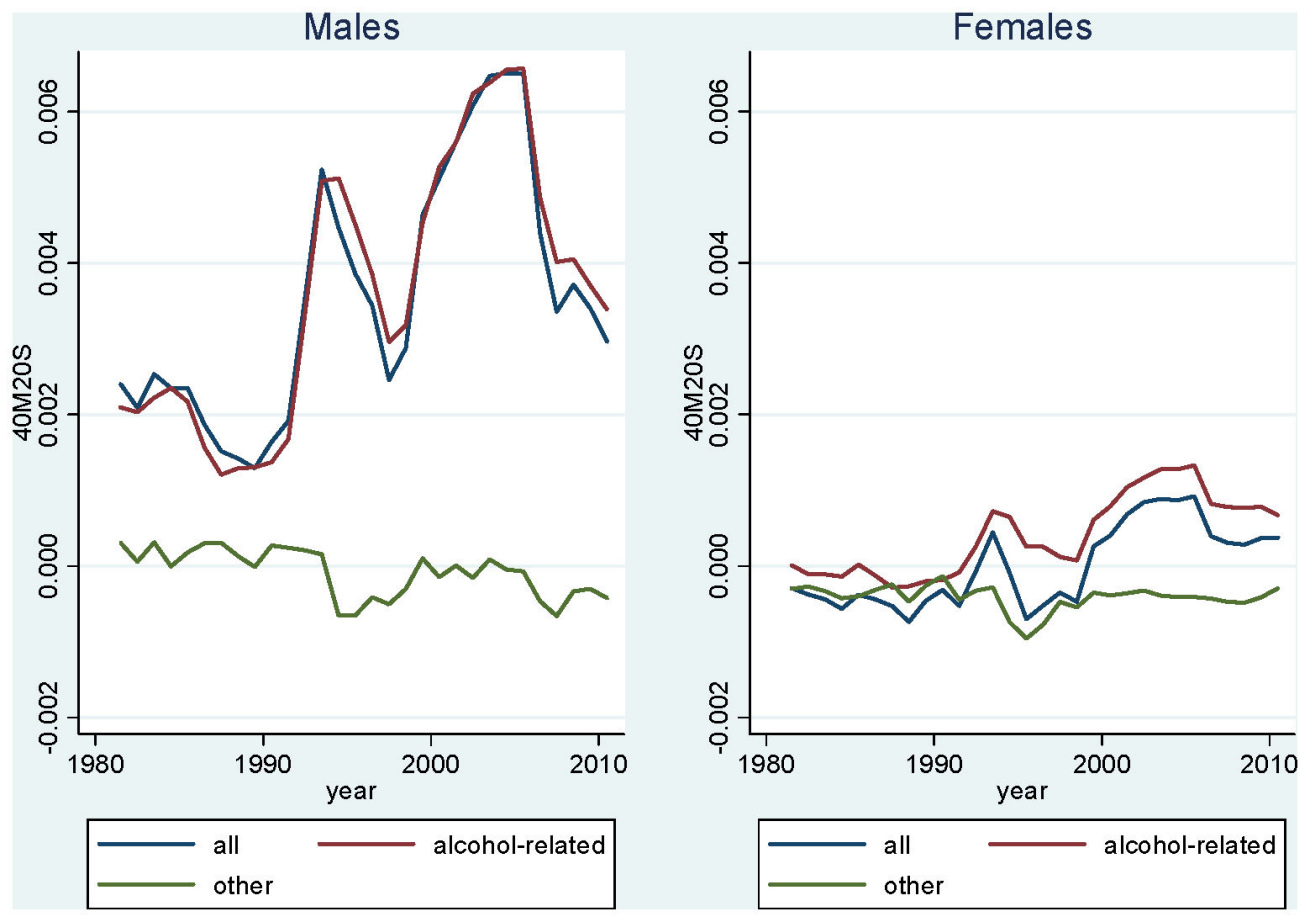
Difference Between Age-Standardized Mortality Rates at Ages 20-59 in Russia and Kyrgyzstan, 1981-2010, All Causes and Strongly Alcohol-Related Causes (Russia Minus Kyrgyzstan). Source: Kyrgyzstan: Official population estimates and death registration tables (Forms 5 [1981-87] and S51 [1988-2010]). Russia: Human Mortality Database [19] for population estimates; Meslé et al. [18] for deaths in 1981-88; Official death registration tables (Forms S51) for deaths in 1989-2010.

Here also, given the importance of external causes and circulatory causes in explaining the gap, it is useful to make the distinction between strongly alcohol-related causes and other causes. Figure 4 shows the contribution of these causes to the Russia-Kyrgyzstan mortality gap between 1981 and 2010. This figure simply shows the difference (Russia minus Kyrgyzstan) in all-cause and cause-specific _40_M_20_^S^. For males, the line for all causes illustrates the gap in all-cause mortality between the two countries, with Russia experiencing an increasing amount of excess mortality during the period. In Figure 4, it appears that excess adult mortality in Russia is basically entirely explained by alcohol-related causes. Other causes contribute virtually nothing – which means that mortality from these causes has been relatively similar in the two countries. As a result, it can be concluded that among males, the mortality gap between Russia and Kyrgyzstan is entirely explained by a set of causes that have been identified as strongly related to alcohol in Zaridze’ study.

For females, alcohol-related mortality is higher in Russia throughout the period, contributing positively to the gap, while mortality is lower for other causes, contributing negatively to the gap. This means that mortality from other causes is actually higher in Kyrgyzstan. The sum of these positive and negative contributions has varied over time. The narrowing of the gap since 1995 and the 1999 cross-over appears to be due to a combination of an increasingly positive role of alcohol-related causes and a diminishingly negative role of other causes.

## Discussion

Cause-specific mortality at adult ages in Kyrgyzstan since the 1980s presents many similarities with Russia, but also some important discrepancies. As in Russia, fluctuations are primarily due to changes in external causes and circulatory causes. As in Russia, alcohol appears to play an important role, as we can see in the mortality decrease during Gorbachev’s anti-alcohol campaign. However, in Russia, mortality from these causes (external, circulatory, and alcohol-related causes) has been higher than in Kyrgyzstan throughout the period, and has increased by a greater amount since the break-up of the Soviet Union. Also, in Kyrgyzstan, a more varied set of causes appear to explain the mortality increase of the 1990s. As a result, the mortality gap that we observe between the two countries is overwhelmingly attributable to external and cardio-vascular causes, and more generally, to causes that have been shown to be strongly related to alcohol consumption.

Given the fact that Kyrgyzstan experienced a more severe economic crisis in the 1990s, and remains substantially poorer than Russia, Kyrgyzstan’s more favorable adult mortality trends cannot be easily explained in macro-economic terms, such as GDP per capita trends, amount of privatization, unemployment, etc. Rather, our cause-specific results suggest that these divergent mortality patterns are likely due to cultural and religious differences between the two countries, and their impact on patterns of alcohol consumption. Indeed, Kyrgyzstan is a predominantly Muslim country, and while excessive alcohol consumption certainly causes a large number of deaths there, as we saw in the previous section, it appears to be a less important feature of Kyrgyzstan’s society, by comparison with Russia [25].

**Figure 5:**
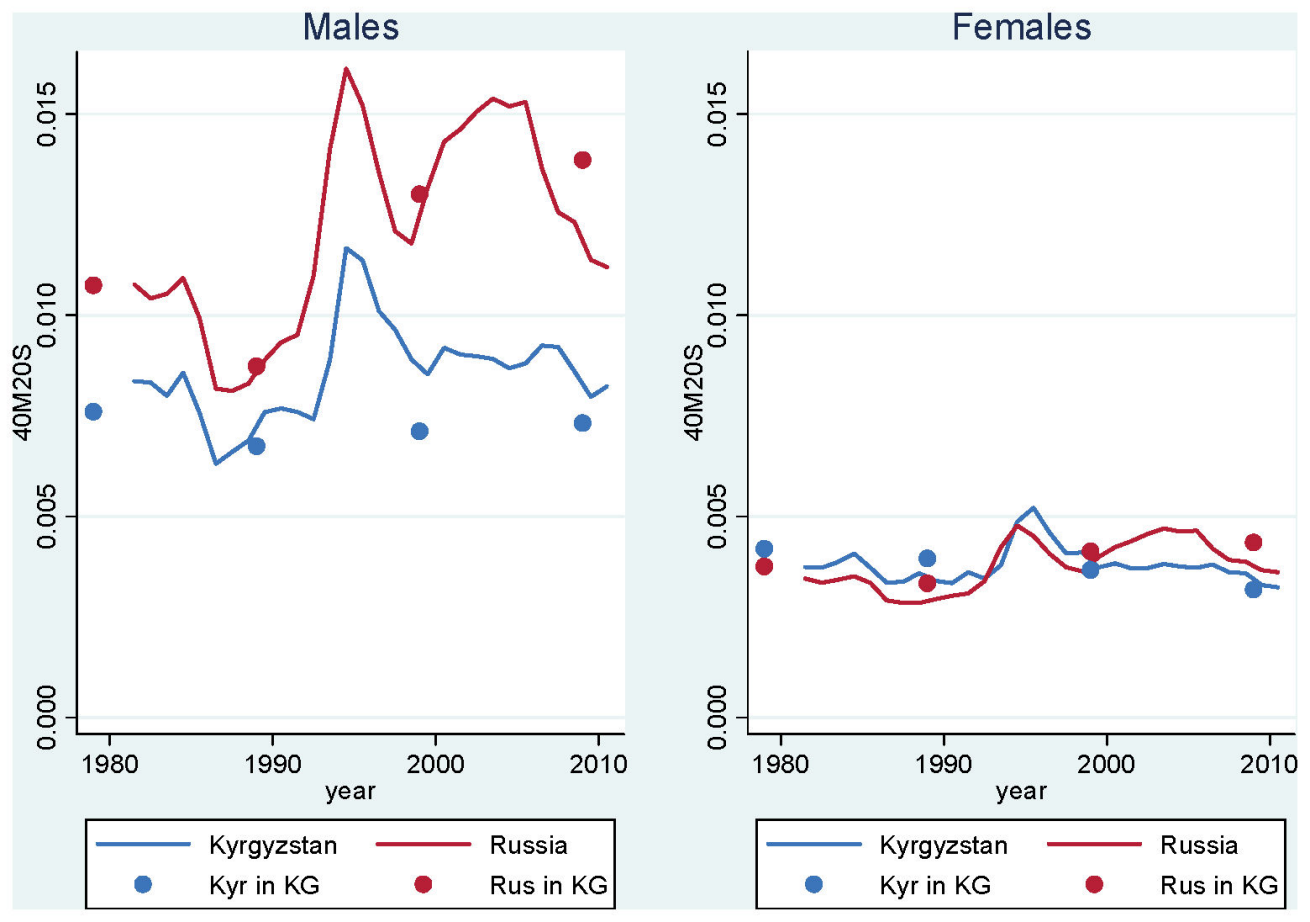
Age-Standardized Mortality Rate at Ages 20-59, Kyrgyzstan (National Level and by Ethnicity), Russia, 1979-2010. Source: Kyrgyzstan: Official population estimates and death registration tables (Forms 4g [1978-79], 5 [1981-87], P44 [1988-89, 1998-99, 2008-09] and S51 [1988-2010]). Russia: Human Mortality Database [19] for population estimates; Meslé et al. [18] for deaths in 1981-88; Official death registration tables (Forms S51) for deaths in 1989-2010.

This interpretation is consistent with mortality patterns by ethnicity in Kyrgyzstan, which show that while ethnic Russians who live in Kyrgyzstan have on average higher socio-economic status than the ethnic Kyrgyz, they experience higher adult mortality, and that this excess mortality is primarily due to alcohol-related causes [15]. Figure 5 updates this result with newly-available information for the year 2009. This figure presents _40_M_20_^S^ for all causes combined in Russia vs. Kyrgyzstan (as in Figure 1), along with the same indicator for the ethnic Kyrgyz and ethnic Russians living in Kyrgyzstan, for the period 1979-2009. (The latter information is available only around census years, which explains the 10-year intervals between estimates.) This information shows that the mortality gap between the two countries is very similar to the mortality gap between members of the two corresponding ethnic groups living in Kyrgyzstan. In particular, ethnic Russians who live in Kyrgyzstan experience adult mortality patterns that are similar to those experienced in Russia. Conversely, Kyrgyzstan’s lower levels of mortality at the national level appear to be driven by lower mortality among the ethnic Kyrgyz. This result further supports the cultural dimension of the mortality gap between the two countries.

If increases in excessive alcohol consumption were directly induced by abrupt socio-economic changes, as has been argued in the case of Russia, one would observe similar, or even larger, mortality increases related to alcohol in Kyrgyzstan. However, alcohol consumption appears to be largely shaped by societal norms [25-27]. The existence of a deeply-embedded “culture of alcohol” in Russia seems to generate so much excess mortality that it wipes out the potential benefits of its more advantageous economic situation, relative to Kyrgyzstan. Conversely, the more muted culture of alcohol in Kyrgyzstan seems to have granted the country with a mortality advantage that appears to overcompensate for its far worse economic outcomes.

The Russia vs. Kyrgyzstan comparison further highlights the overwhelming role that alcohol plays in Russia’s mortality. Although this role is already well documented, the comparison of the two countries provides a richer framework for understanding its root causes. In particular, this comparison highlights the important cultural dimension of alcohol consumption, which is often ignored in explanations of mortality patterns among former Soviet republics. For example, a cross-national study of mortality change in post-communist countries found mortality increases to be related to mass-privatization [3], but the authors did not seek to adjust for baseline patterns of alcohol consumption or related cultural characteristics such as ethnicity. By contrast, in an earlier study, Brainerd [28] found that, among former Soviet republics, the amount of decrease in life expectancy between 1992 and 1994 was largely related to the proportion of Russians in the republic at the beginning of the period. Our results extend Brainerd’s findings and stress the importance of taking ethnic-specific health behaviors and their root causes into account when interpreting cross-national differences in mortality in former Soviet republics.

Levels of adult mortality in Kyrgyzstan, while lower than in Russia, remain an important source of concern. Indeed, today’s levels are similar to those observed in 1981. The cause-specific mortality rates provided in this paper show that policy directed towards prevention and treatment of cardio-vascular diseases, and towards reduction of levels of alcohol consumption, would be particularly beneficial, especially among males. 

## Supporting Information

Table S1 Codes Used for the Calculation of Cause-Specific Mortality in Kyrgyzstan.
(PDF)

Table S2 Codes Used for the Calculation of Cause-Specific Mortality in Russia.
(PDF)
