# Supplementary material for: Divergent Paths for Adult Mortality in Russia and Central Asia: Evidence from Kyrgyzstan
Source: PLoS One. 2013 Oct 8;8(10):e75314. doi: 10.1371/journal.pone.0075314 (PMC3792976; doi:10.1371/journal.pone.0075314)
Supplement: Table S1 — Codes Used for the Calculation of Cause-Specific Mortality in Kyrgyzstan. (PDF) [file pone.0075314.s001.pdf]

Table S1 : Codes Used for the Calculation of Cause-Specific Mortality in Kyrgyzstan

Cause of death	1981-1984	1985-1988	1989-1995	1996	1997-1998	1999	2000-10
All Causes	1-185	1-175	1-175, 206	1-97, 100-175, 196-206	1-97, 100-175, 196-208	1-97, 100-175, 196-209	A00-G99, H60-H95, I00-R00, V01-Y98 (Classes 1-18, 20)
Infectious and Parasitic Diseases	1-44		1-44, 206				A00-B99 (Class 1)
Neoplasms	45-67						C00-D48 (Class 2)
Diseases of the Circulatory System	84-102			84-97, 100-102, 196-205			I00-I99 (Class 9)
Diseases of the Respiratory System	103-114						J00-J99 (Class 10)
Diseases of the Digestive System	115-127						K00-K93 (Class 11)
External Causes	160-185	160-175				160-175, 209	V01-Y98 (Class 20)
Other Causes	68-83, 128-159				68-83, 128-159, 207-208		D50-D89, E00-E90, F00-F99, G00-G99, H00-H59, H60-H95, L00-L99, M00-M99, N00-N99, O00-O99, P00-P96, Q00-Q99, R00-R99 (Classes 3-8, 12-18)
Strongly alcohol-related causes	45-46, 52; 9-13, 43; 103-107, 110-114; 30, 122-123; 126; 92-97; 158-159; 73, 75, 160-185	45-46, 52; 9-13, 43; 103-107, 110-114; 30, 122-123; 126; 92-97; 158-159; 73, 75, 160-175				45-46, 52; 9-13, 43; 103-107, 110-114; 30, 122-123; 126; 92-97; 158-159; 73, 75, 160-175, 209	A15-A19, B15-B19, B90, C00-C15, C32, F10, I20, I24-I28, I30-I52, J00-J18, J30-J39, J47, J60-J99, K70, K74, K85-K86, R00-R99, V01-Y99

Note: The list of strongly alcohol-related causes for the period 2000-2010 slightly differs from Zaridze's list of ICD-10 codes, in order to improve comparability with the earlier Soviet classification.
