# Supplementary material for: Divergent Paths for Adult Mortality in Russia and Central Asia: Evidence from Kyrgyzstan
Source: PLoS One. 2013 Oct 8;8(10):e75314. doi: 10.1371/journal.pone.0075314 (PMC3792976; doi:10.1371/journal.pone.0075314)
Supplement: Table S2 — Codes Used for the Calculation of Cause-Specific Mortality in Russia. (PDF) [file pone.0075314.s002.pdf]

Table S2: Codes Used for the Calculation of Cause-Specific Mortality in Russia

**Cause of death**	**1981-1987**	**1988-1998**	**1999-2010**
All Causes	1-159, 186-195	1-159, 186-195, 206	1-238
Infectious and Parasitic Diseases	1-44	1-44,206	1-55
Neoplasms	45-67	45-67	56-89
Diseases of the Circulatory System	84-102	84-102	115-147
Diseases of the Respiratory System	103-114	103-114	148-164
Diseases of the Digestive System	115-127	115-127	165-179
External Causes	186-195	186-195	229-238
Other Causes	68-83, 128-159	68-83, 128-159	90-114, 180-228
Strongly alcohol-related causes	45-46, 52; 9-13, 43; 103-107, 110-114; 30, 122-123; 126; 92-97; 158-159; 73, 75, 186-195	45-46, 52; 9-13, 43; 103-107, 110-114; 30, 122-123; 126; 92-97; 158-159; 73, 75, 186-195	56-57,65; 9-15, 54; 148, 150-155,160-164; 41-43,173-174; 178; 125-132; 226-228; 97-98, 229-238

Note: The list of strongly alcohol-related causes for the period 1999-2010 slightly differs from Zaridze’s list, in order to improve comparability with the earlier Soviet classification.
